# Risk factors in abdominal aortic aneurysm and in Polish population aortoiliac occlusive disease and differences between them

**DOI:** 10.1038/srep03528

**Published:** 2013-12-18

**Authors:** Joanna Mikołajczyk-Stecyna, Aleksandra Korcz, Marcin Gabriel, Katarzyna Pawlaczyk, Grzegorz Oszkinis, Ryszard Słomski

**Affiliations:** 1 Institute of Human Genetics, Polish Academy of Sciences, Poznan, 60-479, Poland; 2 Department of Vascular Surgery, Poznan University of Medical Sciences, Poznan, 61-848, Poland; 3 Department of Hypertension, Internal Medicine, and Vascular Diseases, Poznan University of Medical Sciences, Poznan, 61-848, Poland; 4 Department of Biochemistry and Biotechnology of the Poznan University of Life Sciences, Poznan, 60-632, Poland

## Abstract

Abdominal aortic aneurysm (AAA) and aortoiliac occlusive disease (AIOD) are multifactorial vascular disorders caused by complex genetic and environmental factors. The purpose of this study was to define risk factors of AAA and AIOD in the Polish population and indicate differences between diseases.

The total of 324 patients affected by AAA and 328 patients affected by AIOD was included. Previously published population groups were treated as references. AAA and AIOD risk factors among the Polish population comprised: male gender, advanced age, myocardial infarction, diabetes type II and tobacco smoking.

This study allowed defining risk factors of AAA and AIOD in the Polish population and could help to develop diagnosis and prevention. Characteristics of AAA and AIOD subjects carried out according to clinical data described studied disorders as separate diseases in spite of shearing common localization and some risk factors.

Abdominal aortic aneurysm (AAA), alongside myocardial infarction and cerebral stroke, are the most common vascular diseases. In recent years, AAA incidence has been on the increase in many countries. The disease affects mainly populations from developed countries which is believed to be connected with the lifestyle associated with high levels of oxidative stress and highly processed food. It should also be noted that AAAs attack mainly the so-called “ageing populations”.

The results of the metaanalysis made by Cornuz and coworkers, including 14 published population studies, showed that 4.1% to 14.2% men and 0.35% to 6.2% women over 60 years of age suffer from aneurysm^1^. Another study performed on a group of 3 million people aged 65–75 indicated the proportion of aneurysm cases at 4.9%^2^. The scale of the phenomenon is a serious health, social and economic problem. Detection of AAA is complicated because it develops without clear symptoms. Moreover, it occurs in elderly, who often suffer from other ailments with serious complications. In Poland, so far, there is no precise statistics showing the number of diagnosed AAAs. Observations made during early AAA diagnosis, carried out at the Medical University in Poznan in 2009–2010 assessed AAA incidence at 2.7%. Studies were performed on a group of 292 men aged 52–89 from Wielkopolska Voivodeship (western Poland).

Aortoiliac occlusive disease (AIOD) is a syndrome caused by lumen narrowing or closing of distal part of the abdominal aorta due to embolism or atherosclerosis. It causes obstruction of distal part of the abdominal aorta and/or iliac arteries and loss of pulse in both lower limbs. It may cause gangrene, lower limb amputation, impotence, cardiovascular complications and death. AIOD is defined as a symptom of atherosclerosis localized only in the abdominal aorta or a symptom of systemic atherosclerosis^3^.

Atherosclerosis is a disorder that affects all people. The disease process varies depending on the exposure to risk factors and genetic predispositions, which, so far, have not been fully understood. The disease begins to develop between 15 and 30 years of life. The process is generally longer than 40 years, and manifests its symptoms by patients between 55–65 years old. According to some researchers, the programming of atherosclerosis begins already in foetal life and it is dependent on mother's exposure to risk factors^4^. The presence of atherosclerotic plaques in the abdominal aorta has been observed already in the second decade of human life^5^. Although majoraty of AIOD patients are over 50, up to 30% of patients are young people^6^. Observations made during the early diagnosis of the aortoiliac occlusive disease at the University of Medical Sciences in Poznan in 2009–2010 determined proportions of the AIOD at 3.4%. Studies were carried out on a group of 292 men aged 52–89 from Wielkopolska Voivodeship.

Despite 30 years of intensive studies, AAA pathogenesis is still unresolved. Molecular background of atherosclerosis is also unexplained. Recently both disorders have been described as multifactorial diseases with a complex genetic background (probably heterogeneity) and influnenced by environmental factors^7^. Risk factors are probably of epigenetic nature and influence the incidence and progression of diseases. Moreover, as a result of different genetic and environmental interactions, they may cause different effects depending on the population. Identification of risk factors would make the diagnostics more effective allowing possibility of detection of diseases in early stages and their prevention by habit changing.

## Results

### Comparison of the AAA and AIOD patients

In the presented study, two groups of patients were compared (the results are shown in Table 1). The obtained results indicate AAA and AIOD patients as two separate population groups. Characteristics of AAA and AIOD subjects carried out according to demographic data and clinical trials (Table 1) revealed that these populations differed with respect to gender, age, body mass index (BMI), myocardial infarction (MI), and hypertension (HT) (differences were statistically significant). No statistically significant differences in terms of complex lipid profile, blood glucose concentration, diabetes type II and smoking habits were recorded.

In the case of both diseases, men constituted a greater part of experimental groups (85.8% in AAA and 74.4% in AIOD patients). The risk of AAA among men was more than twice higher (OR = 2.08; *p* value = 0.0003) when compared with the risk of AIOD. Patients suffering from AAA were, on average, 6 years older than subjects with AIOD (Table 1) which may have been associated with AAA pathology connected with elastolysis. Elastin is a structural protein synthesized mainly during foetal life and babyhood and its half-life is approximately 70 years. Elastolysis is, therefore, a physiological process of aging.

Another factor differentiating the two populations of patients was the body mass index (BMI) defined as the quotient of the weight in kilograms and square of the height in meters (the *p* value < 0.0001, Table 1). The mean and median BMI in patients with AAA were respectively 27.3 and 27.2, which means that the majority in this group were overweight (47.6%) or obese (20.5%) (data not shown). The mean and median BMI values in patients suffering from AIOD were respectively 24.5 and 24.2, which indicated that patients with AIOD did have smaller problems with overweight (29.3%) and obesity (12.4%) than patients with AAA (data not shown). Subjects were considered as hypertensives when their diastolic pressure was above 90 mm Hg or systolic pressure above 140 mm Hg or when they were on antihypertensive therapy. Hypertension occurred significantly more often in patients with AAA (73.5%) in comparison with patients suffering from AIOD (49.7%); (*p* value < 0.0001; OR = 2.81). A similar relationship was observed for myocardial infarction, from which 31.7% AAA patients and 21.7% AIOD patients suffered (*p* value = 0.0043; OR = 1.69). This could attributed to a greater percentage of hypertensive, overweight and obese (68.1%) subjects in AAA patients.

### Risk factors of AAA and AIOD in the polish population

In view of the high percentage of men in both groups of patients, an attempt was made to determine how male gender influenced AAA or AIOD by comparing the proportions of men and women in the analyzed groups with the population of equal numbers of both sexes. Differences between populations of patients with AAA or AIOD and the population, in which the likelihood of women and men was equal, were statistically significant (*p* value < 0.0001), and odds ratios were, respectively OR = 6.04 in the case of AAA and OR = 2.90 in the case of AIOD (Table 2).

The collected data showed that the age of over 66 was a risk factor of AAA, and that of over 60 of AIOD.

It was also examined whether the presence of hypertension differed in patients' populations in comparison with the random population. National Health Survey in Poland Project WOBASZ specifies the proportion of hypertensives (from the same region that the patients groups) at 0.4. The proportion of hypertensives did not differ significantly between the AIOD population and the random WOBASZ population. Comparing patients suffering from AAA with the WOBASZ group, the recorded difference was statistically significant (*p* < 0.0001 and OR = 4.15; Table 2). The obtained results indicated hypertension as a risk factor of AAA in the Polish population.

The percentage of hyperlipidemia defined, according to the Third Report of the National Cholesterol Education Program (NCEP-III) in AAA patients was at 49.0% and in AIOD at 50.5% (Table 1). It is belived that 50% of adults have abnormal lipid profiles. The studied groups of patients did not differ from the random population. It seems that hyperlipidemia is not a significant risk factor of AAA or AIOD in the Polish population. However, it should be stressed that hyperlipidemia is a complex and heterogenic disorder whose effects can be increased by other factors such as hypertension, BMI and smoking habits.

High percentage of people suffering from myocardial infarction (MI) in both populations of patients was also noted. The incidence of MI in a random population is estimated at 0.06^8^, therefore MI occurs much more frequently in patients with studied vascular diseases and differences between patients with AAA and AIOD and random populations are statistically significant. The *p* value in both cases was lower than 0.0001, and the odds ratios were, respectively: OR = 7.43 for AAA and OR = 1.69 for AIOD (Table 2).

Diabetes type II cooccurred with AAA and AIOD in the Polish population. The studied groups of patients did not differ with regard to the above variable. In a random population, the incidence of diabetes type II is assessed at 5.4% (Screen-Pol 2 Project). Therefore it can be said that diabetes type II was significantly more frequent in patients with AAA (11.8%) and patients with AIOD (16.5%) than in the random population (Table 1).

On the basis of the collected data, it was found that the percentage of smokers in patient groups was very high (84.2% for AAA and 82.5% for AIOD, Table 1), Therefore, it was decided to examine whether smoking was a factor predisposing AAA or AIOD in the Polish population. The National Health Survey carried out in Poland (Project WOBASZ) showed that in Wielkopolska Voivodeship, 48% men and 34% women were smokers. Male subpopulation of AAA patients as well as female subpopulation of AAA patients significantly differed from male and female WOBASZ populations The obtained results showed that women smokers were more than six times more vulnerable to AIOD and nearly four times more to AAA and men smokers, almost six times more vulnerable to AIOD and more than seven times more to AAA in comparison with nonsmokers (Table 2). Smoking is a very important risk factor of both diseases.

Phenotypic observations of patients suffering from AAA and AIOD were carried out in accordance with commonly known risk factors of vascular diseases. The exact characteristics of the groups of patients from Table 1 showed statistically significant differences between patient groups. It means that the studied disorders were separate diseases in spite of shearing common localization and some risk factors. This observation is consistent with current views based on the analysis of the AAA induction in animal models. It was demonstrated that it was possible to develop AAA without atherosclerosis and that atherosclerosis did not necessarily lead to the development of aneurysm^9^,^10^.

The obtained results of the performed analysis showed that the AAA risk factors among the Polish population comprised: male gender, advanced age, hypertension, myocardial infarction, smoking hab which i.e. data consistent with reports of other researchers^11^. When compared with random population, AAA patients also comprised higher percentage of individuals suffering from diabetes type II (Table 2). Our research results indicated diabetes as a disease accompanying AAA in the Polish population. This observation differs from previously published reports, which found diabetes, next to the black race, female gender, physical activity and antioxidant rich diets as a factor negatively correlated with aneurysm^11^.

The results determined in previous studies could be attributed to a higher degree of posttranslational glycosylation of matrix proteins as this procces protects them against hydrolysis. However, higher levels of the MMP9 enzyme in plasma in patients with diabetes were detected^12^. It was also reported that glucose blood concentration is related to oxidative stress and induces *MMP2* gene expression in endothelial cells and *MMP9* gene in macrophages^13^. The products of both genes hydrolyse elastin which is the main structural extracellular matrix protein. Elastin hydrolysis is the main cause of abdominal aortic aneurysm development^14^.

AIOD risk factors in the Polish population included: male gender, advanced age, myocardial infarction, diabetes type II and smoking habits. These factors were common to both studied disorders. Differences between them were connected with the fact that the percentage of men in AAA patients was higher and the mean age of patients with AAA was 6 years higher in comparison with AIOD patients. The results indicated the above variables as AIOD risk factors and they were also confirmed by other authors^15^. The performed analyses showed that hypertension, overweight and obesity were not risk factors of AIOD in the Polish population. In addition, these variables differed in the studied groups of patients (Table 1). The obtained results were inconsistent with other published research. It was reported that overweight, obesity and hypertension predispose individuals to developing AIOD^16^. This discrepancy may have resulted from the fact that the above-mentioned published observations were conducted on a group of women. In the presented study, analyses were performed mainly on men as they constituted majority of patients in experimental groups (for AAA – 85.8%; for AIOD – 74.4%; Table 1). Small percentage of women in both populations can be attributed to the protective role of estrogens. A relatively new function of estrogens shows their effect on the production of reactive oxygen species (ROS) in mitochondria. It was found that estrogens reduced ROS concentrations as a result of stimulation of oxidative phosphorylation^17^. In addition, they inhibit production of angiotensin II induced free radicals in vascular smooth muscle cells^18^ and decrease blood pressure by inhibition *ACE* gene transcription in endothelial cells^19^.

Another important risk factor of both diseases is also advanced age. It was shown that male gender and advanced age are correlated with higher levels of the TIMP1 enzyme in plasma^20^. Moreover, overexpression of *TIMP1* gene^21^ and higher levels of the enzyme in patients with AAA^22^ were also reported. McNulty and coworkers demonstrated higher levels of the MMP2 and TIMP2 enzymes in older than in younger human endothelial cell lines^23^. Overexpression of the *TIMP2* gene may affect angiogenesis and stiffness of the blood vessels leading to a higher susceptibility to injury and inflammation^24^. Diseases associated with ageing are also partly explained by age-depended methylation of gene promoters (hypermethylation, i.e. gene silencing and hypomethylation, i.e. gene activation Hypomethylation of promoters was detected by numerous genes in human atherosclerotic tissues and in animal models of the disease^25^. This phenomenon is explained by the tendency of some cells to initiate the atherosclerotic process, particularly in tissues exposed to biomechanical stress, like arteries. Hypermethylations associated with the process of ageing were detected in promoters of vascular homeostasis genes. These processes can be modified by other factors, e. g., smoking habits, diet, or hypertension. Age-depended reduction of histon acetylation was also observed^26^.

Performed observations showed hypertension as a risk factor of AAA in the Polish population. It was found that hypertension was correlated with elevated plasma levels of the MMP9 and TIMP1 enzymes^12^,^20^. Lack of balance between these enzymes caused improper remodeling of the extracellular matrix in the vascular tissue. Other risk factors of AAA comprised overweight and obesity. It was demonstrated that a year after gastrectomy carried out in obese women http://pl.pons.eu/angielski-polski/thelevels of MMP9 enzyme in plasma was reduced^27^.

Of all of the analyzed risk factors, smoking turned out to be the one with the greatest impact on the the occurrence of AAA and AIOD (Table 2). It was shown that individuals who smoked tobacco were 7–13 times more predisposed to AAA^28^. Studies on rat models confirmed the impact of tobacco smoke on the expression of genes. There was an increase in the *TIMP1* gene expression and the *TIMP2* gene expression was lower^29^. Exposure to tobacco smoke resulted in increased *MMP2* gene expression^30^, contributed to *MMP9* gene overexpression^31^ as wall as higher plasma concentration of MMP9 enzyme^12^,^32^ and TIMP1 enzyme^20^,^33^. Smoking is also associated with oxidative stress. Oxidative stress and tobacco smoke change epigenetic modification of chromosomes. Histon acetylation and inflammatory gene promoter methylation contribute to overexpression of inflammatory genes in the epithelial cells^34^. Association between inflammation and vascular diseases was confirmed by numerous research teams. Antioxidant-rich diets can prevent AAA development as was shown on the Ang II induced mice model of AAA^35^. Moreover, diets may influence epigenetic modifications affecting metabolism and homeostasis^4^. It should be stressed that some risk factors such as BMI or smoking, can be eliminated by changing habits. Understanding the role of epigenetic factors in the development of aneurysm and atherosclerosis could allow effective prevention based on proper diets as well as a life style.

In addition, identification the high-risk populations is extremely valuable for patients and helps reduce costs of examinations and possible hospitalization. Defining risk factors would also prevent patients' social exclusion connected with decrease of their quality of life. Therefore, larger studies in different populations may be relevant to better understand the AAA and AIOD pathophysiology and to develop new methods of AAA and AIOD diagnosis and treatment.

## Methods

### Ethics statement

The study was performed in accordance with the 1964 Declaration of Helsinki. The presented study protocol was reviewed and approved by the institutional ethics committee of Poznan University of Medical Sciences. Informed written consent was obtained from all subjects, but data were analyzed anonymously.

### Subjects and methods

In this study, the total of 324 patients affected with AAA and 328 patients affected with AIOD who underwent surgery in the Department of Vascular Surgery of Poznan University of Medical Sciences was included. The groups were selected in cooperation with the Department of Vascular Surgery of Poznan University of Medical Sciences. AAA was defined as a focal dilation of the abdominal aorta at least 50% larger than the adjacent suprarenal segment. The group of patients with AAA selected by the physicians was represented by patients with aneurysm of 20–125 mm in diameter, located in a distal part of the abdominal aorta and iliac arteries. Familial AAA cases and patients with aneurysm characterized by inflammation, dissection or emerged as a result of trauma were excluded from the study. The AIOD group included patients with clinical symptoms of ischemia in III and IV degree (Fontaine's) diagnosed by both anatomical and symptomatic criteria. All studied individuals underwent complete clinical examination to exclude subjects suffering from symptoms of any inflammatory or neoplastic diseases as well as those with lung emphysema, formation of cysts, or cystomas in parenchymatous organs. Chronic renal failures or cerebrovascular disease also disqualified from the study. Data concerning medical records, medication history and smoking habits were collected based on detailed questionnaire. Lipid fractions were measured after overnight fasting. All subjects underwent Doppler ultrasound scanning examination. Aortic diameter was measured below the origin of renal arteries. To define AAA and AIOD risk factors in the Polish population, previously published population groups were used as references [ref. 8; Screen-Pol 2 Project; National Health Survey in Poland Project WOBASZ].

### Statystical analysis

Statistical analysis was performed using GraphPad Prism and Statistica software. For the comparison of groups of patients and to indicate risk factors, nonparametric tests and exact Fisher test, χ^2^-test and χ^2^-test with Yates correction were used. The odds ratio (OR) with 95% CI was determined. A *p* value < 0.05 was considered to indicate statistical significance. All tests were two-tailed.

## Author Contributions

J.M.S. and R.S. wrote the manuscript text; M.G., K.P. and G.O. examinated and selected patients for study, J.M.S., A.K., M.G., K.P. and G.O. collected material for study; J.M.S. performed statistical analysis. All authors reviewed the manuscript.

## Figures and Tables

**Table 1:** Demographic and clinical characteristics of patients groups

Variable	AAA n = 324	AIOD n = 328	P value	OR (95% CI)
Gender/women	14.2%	25.6%	.0003	2.08(1.40–3.10)
/men	85.8%	74.4%		
Age (mean ± SD)	66.8 ± 8.29	60.3 ± 8.29	<.0001	-
BMI (mean ± SD)	27.3 ± 3.94	24.5 ± 4.50	<.0001	-
Total cholesterol [mmol/L] (mean ± SD)	5.3 ± 1.45	5.3 ± 1.37	ns	-
HDL-Chol [mmol/L] (mean ± SD)	1.2 ± .36	1.2 ± .44	ns	-
LDL-Chol [mmol/L] (mean ± SD)	3.3 ± 1.27	3.3 ± 1.13	ns	-
TGC [mmol/L] (mean ± SD)	1.7 ± 0.84	1.7 ± .87	ns	-
Glucose [mmol/L] (mean ± SD)	6.0 ± 1.60	6.4 ± 2.60	ns	-
Myocardial infarction	31.7%	21.6%	.0043	1.69(1.18–2.42)
Hypertension	73.5%	49.7%	<.0001	2.81(2.00–3.94)
Hyperlipidemia	49.0%	50.5%	ns	-
Diabetes type II	11.8%	16.5%	ns	-
Smoking habit	84.2%	82.5%	ns	-

P value < .05 means that difference between tested groups is statistically significant. Abbreviations: SD standard deviation, OR odds ratio, ns non-significant, HDL-Chol high density lipoprotein, LDL-Chol low density lipoprotein, TGC triglicerides. BMI body mass index.

**Table 2:** Risk factors of AAA i AIOD in the Polish population

	AAA vs*.* random population		AIOD vs. random popopulation	
Variable	P value	OR	P value	OR
Gender (men vs. women)	<.0001	6.04	<.0001	2.90
Hypertension	<.0001	4.15	ns	-
Hyperlipidemia	ns	-	ns	-
Myocardial infarction	<.0001	7.43	<.0001	4.29
Diabetes type II	.0054	2.41	<.0001	3.46
Smoking habits: women	.0100	3.86	<.0001	6.36
Smoking habit: men	<.0001	7.65	<.0001	5.97

## Notes

The study was supported by Polish Ministry of Science and Higher Education [N N402 471437; N N403 209635] and by European Social Fund [special award for Joanna Mikołajczyk-Stecyna].
